# *Parascaris* spp. eggs in horses of Italy: a large-scale epidemiological analysis of the egg excretion and conditioning factors

**DOI:** 10.1186/s13071-021-04747-w

**Published:** 2021-05-08

**Authors:** Antonio Scala, Claudia Tamponi, Giuliana Sanna, Giulio Predieri, Luisa Meloni, Stephane Knoll, Giampietro Sedda, Giorgia Dessì, Maria Grazia Cappai, Antonio Varcasia

**Affiliations:** 1 Dipartimento di Medicina Veterinaria, Università di Sassari, Sassari, Italy; 2 ACME s.r.l., Reggio Emilia, Italy

**Keywords:** *Parascaris*, Horses, Italy, Epidemiological survey, Treatments, Ascariosis

## Abstract

**Background:**

Equine ascariosis, caused by *Parascaris* spp., is a worldwide endoparasitic disease affecting young horses in particular. Despite the great number of horses reared in Italy, large-scale epidemiological surveys dealing with ascariosis prevalence in the country are not reported in the current literature. For this reason, the present survey aims to describe, for the first time, the spread and infestation of *Parascaris* spp. in a large population of Italian horses (6896 animals) using faecal egg counts, and further to identify risk factors associated with ascarid egg shedding.

**Methods:**

Individual rectal faecal samples collected during routine veterinary examinations were used and *Parascaris* spp. prevalence was tested against the animal’s age, sex, housing conditions, geographic provenance as well as the respective sampling season.

**Results:**

Among the examined stables, 35.8% showed at least one horse to be positive for *Parascaris* spp. eggs and an overall prevalence of 6.3% was found. Ascariosis rates tended to decrease significantly with age and, proportionally, 80.0% of the recorded *Parascaris* spp. eggs were found in 0.7% of the examined animals. Statistically significant differences among prevalence rates were found between the different geographic areas of provenance and prevalence was found to be higher in horses reared outdoors compared to those raised indoors. Analysis of data based on sex and season did not show any significant differences. Despite the lower prevalence found compared to other European countries, ascariosis was concluded to represent a significant health challenge for horses reared in Italy, especially foals. Age (foals and yearlings) and outdoor rearing were identified to be significant risk factors for *Parascaris* spp. egg shedding. Furthermore, the relevance of the infected horses over 6 years of age should not be underestimated as these represent a significant source of contamination for younger animals.

**Conclusions:**

The development of improved treatment protocols based on regular faecal examination combined with follow-up assessment of the efficacy of integrated action plans would prove beneficial in regard to animal health and anthelmintic resistance reduction in the field.

**Graphic Abstract:**

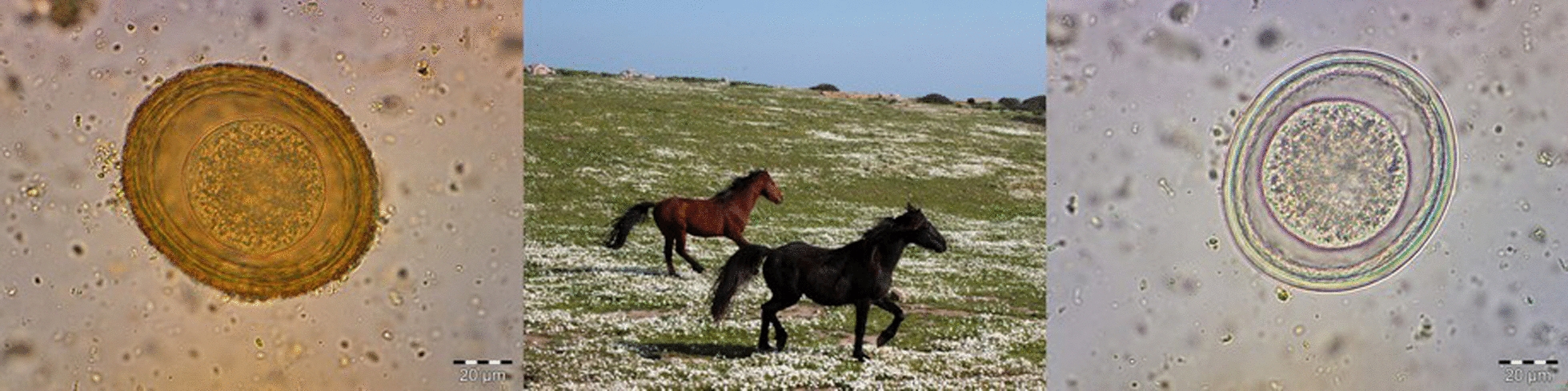

## Background

Equine ascariosis is a worldwide endoparasitic disease caused by *Parascaris* spp. that predominantly affects the health status of young horses [1, 2]. In foals, these parasites can be found in up to 100% of cases [3, 4] and, according to its severity and stage, is characterised by nasal discharge, coughing, a rough or bristly hair coat, slow growth and occasional small intestine rupture following the heavy accumulation of worms [5–7]. Nevertheless, the main clinical finding related to *Parascaris* spp. infection is small intestinal impaction [6, 7].

Typically, ascariosis is defined as an infection caused by *Parascaris equorum*; however, in the USA, Switzerland and Sweden, *Parascaris univalens* has recently been recognized as the dominant species affecting horses [8–10]. In any case, the two species are considered morphologically identical and can only be distinguished through karyotyping; *P. univalens* possesses one pair of germ line chromosomes with respect to the two of *P. equorum* [9, 11]. Additionally, recent mitochondrial genome analysis revealed these two to be very closely related and may represent the same species after all [12].

The life cycle of *Parascaris* spp. initiates with the ingestion of parasite eggs containing infective L2 larvae (larvae develop within 9–14 days after egg excretion within the faeces of infected horses) by a suitable host [7]. After hatching in the host’s intestine, larvae embark on a migration following the hepato-tracheal migratory route before finally returning to the small intestine [13, 14]. Adult development occurs in the jejunum where females will start laying eggs within about 10–15 weeks of infection [5, 15, 16].

Mature *Parascaris* spp. females are particularly fertile, which can lead to high faecal egg counts (FEC) and significant soil and pasture contamination (requiring adequate management), even in the case of limited parasite burden [17, 18]. Overall, a single infected horse has been reported to be able to output upwards of 50 million eggs per day [19]. Furthermore, eggs of *Parascaris* spp. can survive in the external environment (pastures, paddocks, boxes, etc.) in excess of 1 year [13, 18, 20].

Foal ascariosis control programs are based on year-round monthly rotational anthelmintic treatment with benzimidazole (e.g. fenbendazole—FBZ), tetrahydropyrimidine (e.g. pyrantel—PYR) and macrocyclic lactones (e.g. ivermectin—IVM and moxidectin—MOX) [21]. In Italy, the treatment of foals is predominantly carried out using IVM and PYR starting from 2 months of age and is repeated every 2 months up to 1 year of age [22].

In *Parascaris* spp., anthelmintic resistance (AR) to macrocyclic lactones, probably due to intensive use of these drugs, has been widely reported since 2002 [23–25]. Additionally, recent reports have suggested AR to pyrantel salts and benzimidazole drugs to be present as well [1, 26, 27]. In Italy, AR to PYR and IVM in ascarids in horses has been reported since 2009 [22].

Recently, a survey on the therapeutic plan applied by Italian horse breeders for the control of ascariosis highlighted that anthelmintic treatment is administered without prior faecal examination in 85.3% of cases, and in 57.8% with a dosage based solely on a visual weight estimation of the animal. Furthermore, only 21.3% of the farmers generally removed horse faeces from pastures as part of ascariosis prophylaxis [28].

Despite the large number of horses (154,505 individuals) reared in Italy, to the best of our knowledge, no large-scale epidemiological surveys dealing with ascariosis prevalence in horses are reported in the present literature. Moreover, studies which do graze this subject are mostly related to anthelmintic efficacy trials [22, 29].

Against this background, the present survey aims to describe, for the first time, the spread and infestation of *Parascaris* spp. in a large population of horses throughout Italy using faecal egg counts (FEC), and further to identify risk factors associated with ascarid egg shedding.

## Methods

### Sampling and coprological examination

The study population for this research had been defined in a parallel study evaluating the excretion of gastrointestinal strongyle eggs [30].

A total of 6896 randomly selected horses, ranging from 6 months to 36 years old, were examined through coproscopic analysis for the presence of ascarid eggs between 2015 (n. 4164) and 2016 (n. 2732). Animals from 548 different stables located throughout all regions and islands of Italy were included.

Individual rectal faecal samples collected during routine veterinary examinations were sent, vacuum-packed, in refrigerated containers, to the parasitology laboratory of the Department of Veterinary Medicine of the University of Sassari through an express courier within 3 days after collection.

Each faecal sample was accompanied by a form filled in by the sampling veterinarian with the horse’s data including age, sex, housing conditions and stable. Coprological examination was carried out through the modified McMaster method as described by Raynaud [31], using a sodium chloride (NaCl) supersaturated solution (specific gravity = 1.2) for flotation and an egg detection limit of 15 eggs per gram (EPG) of faeces. *Parascaris* spp. eggs were identified by morphology [7].

### Statistical analysis

Data were collected on a spreadsheet (Microsoft Excel^®^) and subsequently processed using Minitab^®^ (Minitab, Inc., State College, Pennsylvania, USA) and Epi Info^®^ 6.0 (Centers for Disease Control and Prevention [CDC]/World Health Organization [WHO], Atlanta, GA, USA) software. For elaboration, χ^2^ test, χ^2^ test for trend, Pearson correlation, Mann-Whitney test, Kruskal–Wallis test and calculation of odds ratio (OR) were performed. Results were considered statistically significant at a *P*-value lower than 0.05 (*P* < 0.05).

Statistical analyses were performed based on various attributes of the monitored horses including age groups (6–11 months, 1 year, 2 years, 3 years, 4–5 years, 6–10 years, > 10 years of age); sex; sampling season (winter, spring, summer, autumn) and housing condition (box, outdoor, indoor/outdoor). Animals were stratified into five classes according to their parasite EPG levels: (1) ≤ 50 EPG; (2) between > 50 and 200 EPG; (3) between 200 and 500 EPG; (4) between > 500 and 1000 EPG; (5) > 1000 EPG.

Data were also processed according to the animals’ geographic provenance as follows: Zone 1-Northern Italy (Piedmont, Lombardy, Trentino Alto Adige, Friuli Venezia Giulia, Veneto, Emilia Romagna and Liguria); Zone 2-Central Italy (Tuscany, Umbria, Marche, Latium, Abruzzi, Molise); Zone 3-Southern Italy (Campania, Basilicata, Apulia, Calabria and Sicily).

The mean intensity (MI) was calculated as the arithmetic mean of EPG values on the total number of infected horses in their respective category.

## Results

Among the examined stables, 35.8% showed at least one horse to be positive for *Parascaris* spp. eggs (95% CI 31.9–39.9) and the overall prevalence for all examined horses was 6.3% (435/6896; 95% CI 5.8–6.9). An EPG mean of 40.0 ± 454.2 and a MI of 634 ± 1702.9 was obtained.

Kolmogorov–Smirnov normality test showed the EPG values attained from the examined horses not to be normally distributed (KS = 0.472; *P* = 0.010).

Data processed by year showed prevalence rates of 6.8% (95% CI 6.1–7.6) and 5.6% (95% CI 4.8–6.5) with EPG means of 44.8 ± 512.4 and 32.64 ± 326.15 (Mann–Whitney test: U = 14,429,342, *P* = 0.386) and MI of 659 ± 1899 and 587 ± 1264 (*U* = 33,661, *P* = 0.794) for 2015 and 2016, respectively. A statistically significant difference in overall prevalence was found between both years (χ^2^ =  4.2412, *P* = 0.0394).

Data processed according to age groups for 5065 horses are shown in Table 1. Prevalence rates tended to decrease significantly with age (χ^2^ = 386.202, df= 6, *P* < 0.001) as well as EPG means (Kruskal–Wallis test: *H* = 491.09, *P* < 0.001), MI of EPG (*H* = 25.79; *P* < 0.001) and OR values; the correlation between age and EPG levels was in effect negative (Pearson Correlation *r* = − 0.079; *P* < 0.001).

**Table 1:** Prevalence rates, mean eggs per gram (EPG) value and mean intensity (MI) of EPG reported for different age groups and their statistical analysis (*P*-value < 0.001)

Age groups (year)	Examined	Prevalence %	Odds ratio (95% CI)	Mean EPG (SD)	MI EPG (SD)
< 1	128	36.7	1.00 (0.67–1.49)	546.0 (1978.0)	1486.6 (3061.6)
1	365	17.3	0.36 (0.27–0.48)	45.5 (171.9)	263.3 (339.5)
2	339	10.9	0.21 (0.15–0.30)	9.9 (39.2)	90.8 (82.9)
3	351	14.5	0.29 (0.21–0.40)	54.5 (229.5)	375.1 (495.9)
4–5	565	7.8	0.15 (0.11–0.21)	18.8 (130.2)	241.0 (409.4)
6–10	1447	3.0	0.05 (0.04–0.07)	15.4 (204.9)	505.5 (1076.5)
> 10	1870	1.2	0.02 (0.01–0.03)	10.7 (216.8)	908.2 (1824.5)

EPG: eggs per gram; MI: mean intensity; SD: standard deviation; 95% CI: 95% confidence interval

Proportionally, 80.0% of the recorded *Parascaris* spp. eggs were found in 49 horses, representing 0.7% of the total examined animals and having EPG values between 750 and 19,065. Moreover, 48.5% (95% CI 0.36–0.63) of these horses were younger than 1 year of age.

Analysis of data based on sex (on a subset of 5137 animals: 2366 females and 2771 males) did not show any significant differences (χ^2^ = 0.1663; *P* = 0.683) (Table 2). Neither did Mann–Whitney comparison between EPG means (*U* = 6,088,128; *P* = 0.851) and MI EPG (*U* = 20,702; *P* = 0.074) show significantly different values in females compared to males.

**Table 2:** Prevalence rates, mean eggs per gram (EPG) EPG value, mean intensity (MI) of EPG and odds ratio (OR) reported for different seasons, sex, areas and housing conditions

	Seasons				Sex		Areas			Housing condition		
	Winter	Spring	Summer	Autumn	Male	Female	Zone 1	Zone 2	Zone 3	Box	Outdoor	Indoor**/**outdoor
Examined	1937	2083	1230	1646	2771	2366	4118	1878	898	2383	949	2364
Prevalence %	6.5	6.8	5.1	6.4	5.5	5.8	6.7	5.3	3.7	4.4	8.1	4.4
Mean EPG Mean	45.2	30.0	28.0	55.3	29.4	29.3	40.9	32.1	52.1	22.1	55.6	39.2
MI EPG	700.5	440.3	549.3	867.5	536.3	510.2	610.6	184.6	214.6	506.4	156.8	115.5
Odds ratio (95% CI) (OR)	1.00 (0.83–1.2)	1.06 (0.89–1.26)	0.78 (0.60–1.01)	0.99 (0.81–1.21)			1.00 (0.88–1.13)	0.77 (0.62–0.95)	0.53 (0.37–0.75)	1.00 (0.82–1.22)	1.94 (1.52–2.47)	1.01 (0.83–1.23)
*P*-value	*P* = 0.509						*P* = 0.0008			*P* < 0.001		

EPG: eggs per gram; MI: mean intensity; 95% CI: 95% confidence interval

Seasonal prevalence rates did not differ significantly (χ^2^ = 0.437; df = 3; P = 0.509), nor did the mean EPG levels (*H* = 53.09; *P* = 0.272) and EPG MI values (*H* = 1.79; *P* = 0.618) show any significant differences. Results stratified by sampling seasons are reported in Table 2.

Results obtained through stratification based on the geographic provenance (Table 2) showed significant differences between the prevalence rates for the three different areas, increasing from south to north (χ^2^ = 14.155; df = 2; *P* = 0.0008). Non-parametric Kruskal–Wallis test showed no significant differences with respect to the EPG means (*H* = 5.27; *P* = 0.072) and EPG MI (*H* = 1.79; *P* = 0.617) in the three investigated areas.

Data stratification related to 5696 horses based on housing type highlighted significant differences in *Parascaris* spp. prevalence (χ^2^ = 23.119; df = 2; *P* < 0.001), with the highest prevalence rates found in horses raised outdoors (8.1%). Comparison between the EPG means found in horses with different lifestyles was also significant through Kruskal–Wallis test (*H* = 53.09; *P* < 0.001). The highest EPG means (55.6 EPG) were found in horses reared outdoors, while the highest MI EPG were recorded in box-raised horses (506.4 EPG). Results related to housing type are reported in detail in Table 2.

Data processed according to EPG classes are shown in Table 3. Prevalence rates as well as OR values tended to decrease significantly in the highest EPG levels (χ^2^ = 16,231.2; df = 5; *P* < 0.0001).

**Table 3:** Prevalence rates reported according to classes of eggs per gram (EPG) for the 435 horses shedding *Parascaris* spp. eggs (*P*-value <  0.0001)

EPG classes	Positives horses	Prevalence %	Odds ratio (95% CI)
≤ 50 EPG	135	31.0	1.00 (0.79–1.25)
> 50 ≤ 200 EPG	124	28.5	0.89 (0.71–1.12)
> 200 ≤ 500 EPG	77	17.7	0.48 (0.37–0.62)
> 500 ≤ 1000 EPG	39	9.0	0.22 (0.16–0.31)
> 1000 ≤ 2000 EPG	32	7.4	0.18 (0.12–0.26)
> 2000 EPG	28	6.4	0.15 (0.10–0.22)

EPG: eggs per gram; 95% CI: 95% confidence interval

## Discussion

The present study reports, for the first time, results of an epidemiological survey on *Parascaris* spp. eggs in Italian horses, including over 6000 animals from all over the country. More than one-third of the examined stables (38.5%) tested positive for *Parascaris* spp. eggs, which is lower than the prevalence reported in the literature for Finland (47%) [32], the UK (50%) [33] and Kentucky (86%) [34]. The overall *Parascaris* spp. prevalence (6.3%) found in horses screened in this survey is lower than those reported in similar studies carried out in other countries [1, 3, 32, 33, 35–38], but higher than that reported in Germany [39], as shown in Table 4. However, the prevalence of *Parascaris* spp. eggs found in foals (36.7%), which allows for a better comparison of the spread of infestation of these parasites, aligns with the results of a previous study carried out in the UK (38%) [33]. This being said, the prevalence rate of *Parascaris* spp. in yearlings reported in the present study (17.3%) is higher than the 4% reported by Relf et al. [33]. Furthermore, the prevalence of egg shedding reported in foals in our survey is higher than that reported in Germany (2.9% in horses up to 2 years of age) [39] where the parasitic pressure seems to be particularly low compared to other studies, including this one; this is probably due to the management of outdoor conditions with particular regard to the horse pasture safety and hygiene management in a broad sense [40]. Considerably higher prevalence of egg shedding was found in Normandy, France (30.5% with EPG means of 388) [3] and Finland (50% with EPG means of 360) [2]. Lastly, a study carried out on horses up to 2 years of age in Cuba reported a prevalence of *Parascaris* spp. of 26.7% [38], while the prevalence found in our study was reported to be 17.7% for the same age category.

**Table 4:** Overall *Parascaris* spp. prevalence reported in different countries

Country	Prevalence (%)	Examined animals	References
Italy	6.3	6896	Present study
Australia	58.3	252	[1]
France	30.5	455	[3]
Finland	11.5	139	[32]
United Kingdom	9	1221	[33]
Ethiopia	43.8	384	[35]
Iran	20.8	375	[36]
Palestine	15.6	435	[37]
Cuba	10	396	[38]
Germany	3	400	[39]

Unfortunately, further direct comparison between the present and other studies was not possible due to a lack of data uniformity.

Considering the *Parascaris* spp. mean FEC value of 209 EPG reported in the UK for foals younger than 1 year [33], a much higher value was found in Italy in the present study (EPG mean 546). In accordance with the results reported by Relf et al. [33], the present EPG mean does decrease with increasing age. Regardless, despite this decreasing trend, the recorded MI in older horses should not be underestimated as this could represent a significant source of environmental contamination and may pose a risk to younger animals. Especially foals within the youngest age group could be at risk as they are likely to ingest ascarid eggs while suckling the mare and exploring their immediate environs [13]. Besides, ascaridiosis is occasionally diagnosed in immunocompetent adult horses as well, even if such infections are typically observed on properties where foals also reside and do show overall low FEC values (< 50 EPG) [41].

In any case, prevalence rates and EPG values reported in this survey agree with previous studies in which ascarids are reported to be more common in horses younger than 2 years [42, 43]. Indeed, *Parascaris* spp. are widely recognized as the most common and pathogenic parasite in foals and yearlings [15, 25]. Most likely, this is due to the significant age-dependent and active immunity of foals, which seems to develop at around 6 months of age [19]. In fact, a bimodal pattern of *Parascaris* spp. egg shedding has been reported with a primary peak at 3–4 months followed by a secondary more contained peak at 8–10 months of age [44].

Despite the statistically significant differences between the overall prevalence rates of *Parascaris* spp. eggs recorded in 2015 and 2016 (6.8% in 2015 vs 5.6% in 2016; χ^2^ = 4.2412; *P* = 0.0394), environmental contamination levels seem to be uniform during these two periods as no significant differences in mean EPG and MI values (*P* < 0.05) could be pointed out. The lower prevalence recorded in 2016 (5.6% vs. 6.8% in 2015) could be explained through differences in climatic conditions in Italy observed between the two years. In 2016, a severe drought was recorded [45, 46], possibly leading to lower pasture and paddock ascarid egg loads [43].

It is important to note that the proportion of the animals found to be shedding 80.0% of the total eggs in this research (0.7%) is lower than those reported in other countries [33]. Regardless, such *Parascaris* spp. “high shedders” do not have the same epidemiological importance as those shedding strongyle eggs [47]. In fact, while faecal examination is adequate for the detection of ascarid eggs shedding in general, the magnitude of quantitative egg counts is not correlated to the size of the worm burden [47, 48].

Unfortunately, selective treatment strategies widely recommended for strongyle control in adult horses, with treatments based on FEC, cannot be applied in juvenile horses. In these animals (foals), which are all considered susceptible and more prone to ascarid infections, an age-defined approach is recommended [13]. At that stage, egg counts merely reflect the foal’s age rather than the actual infection status. Furthermore, leaving a proportion of foals on a given farm untreated is risky and ill-advised as cases of ascarid-associated colic most often occur in previously untreated foals between 4 and 7 months of age [7].

Regarding the seasonal data, in line with previous studies carried out in Europe [32, 39] and the USA [15], no statistically significant differences between the prevalence rates as well as the EPG means were found. Absence of seasonality of the ascarid burden in horses from Italy could be related to the high resistance of the infective eggs in the environment [18, 20] and the Mediterranean climate characterizing Italy. Indeed, significant variations in seasonal trends of ascariosis have been reported in horses raised in more arid climates such as Saudi Arabia [49].

In line with previous studies, no significant differences were found between the prevalence rates, EPG means and MI between sexes [37, 50–52]. The vast majority of positive animals in this study consisted of young horses managed in a uniform manner, independently of their respective sex (fillies or colts). Therefore, any significant correlation between gender and prevalence and intensity of infection reported by Imani-Baran et al. [36] is, as stated by Relf et al. [33], probably due to variations in management strategies rather than differences in susceptibility or receptivity between fillies and colts.

Results regarding the animals’ housing suggest keeping animals outdoors (compared to horses being kept in stables) to be an important risk factor for the occurrence of ascariosis, as previously reported [2, 33, 38]. In this regard, hygienic management of manure, which is applied on a daily basis for stabled horses, may explain the reduced ascarid burden reported in the literature [2]. However, our results point to stabled horses having higher MI and mean EPG values (506.4 EPG) and thus requiring appropriate manure management protocols to reduce environmental contamination of confined spaces such as boxes.

Although very little is known regarding effective procedures for the reduction of *Parascaris* spp. pressure at present, management of pastures, paddocks and stalls can play a critical role in ascariosis epidemiology. Furthermore, conditions required to reduce ascarid contamination may be substantially different from those known to be effective against strongyles [7]. Next, daily management of the manure in boxes where foals and mares are kept year-round could prove an effective control strategy considering the resistant nature of ascarid eggs. Lastly, little is known regarding which molecules (and their relative dosage) may be appropriate for the safe removal or inactivation of equine ascarid eggs from the environment [7, 13].

## Conclusions

In conclusion, results of the present survey show, despite the lower *Parascaris* spp. prevalence found compared to other European countries, ascariosis to represent a health challenge for horses reared in Italy, especially young animals. Furthermore, the relevance of the MI attained from horses over 6 years of age should not be underestimated as these animals represent a possible source of continuous re-infestation responsible for significant environmental contamination and horizontal transmission. Thus, these horses can be considered “egg shedders”, posing a severe health challenge for young animals. Finally, housing type (specifically outdoor rearing) and geographical location should be implemented as risk factors within *Parascaris* spp. monitoring and control programs for Italian horses, with a specific focus on reduction of risk exposure.

## Data Availability

The datasets used and/or analysed during the current study are available from the corresponding author on reasonable request.
